# 

**Published:** 1885-10

**Authors:** 


					﻿io cts. a copy.
OCTOBER. 1885.
$ i .00 per year.
HALES®
eJOVRNAL'J
HE Al TH
ESTABLISHED 1854
^■3 2
o/H- IO
OFFICE, 75 & 77 BARCLAY STREET, NEW YORK
Entered as Second-class Matter at the Post-Office, New York.
				

